# Holographic Performance of Azo-Carbazole Dye-Doped UP Resin Films Using a Dyeing Process

**DOI:** 10.3390/ma12060945

**Published:** 2019-03-21

**Authors:** Kenji Kinashi, Isana Ozeki, Ikumi Nakanishi, Wataru Sakai, Naoto Tsutsumi

**Affiliations:** 1 Faculty of Materials Science and Engineering, Kyoto Institute of Technology, Kyoto 606-8585, Japan; wsakai@kit.ac.jp; 2 Master’s Program of Innovative Materials, Graduate School of Science and Technology, Kyoto Institute of Technology, Matsugasaki, Sakyo, Kyoto 606-8585, Japan; isana.oz.0611@gmail.com (I.O.); m7616023@edu.kit.ac.jp (I.N.)

**Keywords:** dyeing, unsaturated polyester resin, azobenzene, hologram, aqueous dispersion

## Abstract

For the practical application of dynamic holography using updatable dyed materials, optical transparency and an enlarged sample size with a uniform dispersion of the dye and no air bubbles are crucial. The holographic films were prepared by applying a dyeing method comprising application, curing, dyeing, and washing to an unsaturated polyester (UP) resin film. The unsaturated polyester (UP) resin film with high optical transparency was dyed with a 3-[(4-cyanophenyl)azo]-9*H*-carbazole-9-ethanol (CACzE) (azo-carbazole) dye via the surfactant, polyoxyethylene (5) docosyl ether, in an aqueous solution. The amount of dye uptake obtained via the dyeing process ranged from 0.49 to 6.75 wt.%. The dye concentration in the UP resin was proportional to the dye concentration in the aqueous solution and the immersion time. The UP resin film with 3.65 wt.% dye exhibited the optical diffraction property *η*_1_ of 0.23% with a response time *τ* of 5.9 s and a decay time of 3.6 s. The spectroscopic evaluation of the UP resin film crosslinking reaction and the dyeing state in the UP resin film are discussed. Furthermore, as an example of its functionality, the dynamic holographic properties of the dye-doped UP resin film are discussed.

## 1. Introduction

Polymer-based dynamic holographic films have attracted considerable attention as a next-generation 3D display technology [1,2,3,4,5,6,7]. Optical transparency and an enlarged sample size with a uniform dispersion of the dye and no air bubbles are the minimum requirements for the practical application of dynamic holograms [3]. In our 2016 report [3], we proposed insightful scientific research to allow transparency in the visible region for the azo-carbazole analog based on spectroscopic and holographic optical perspectives. Melt-pressed poly(methyl methacrylate) (PMMA) films dispersed with azo-carbazole dyes give high optical transparency with a uniform coloring state and no air bubbles. Making an enlarged size film via melt-press on a laboratory scale, however, has some limitations. Notably, the fabrication of the large sample size would be challenging. Therefore, the development of a new method to replace the melt-press method is necessary.

Dye-doped polymeric films possess well-known properties, such as the manipulation and detection of light. Therefore, these films have been used as optoelectronic devices, such as organic dye lasers, organic light-emitting diodes (LED), dye photodetectors, dye-sensitized solar cells, and displays [8,9,10,11]. Solution processes such as spin-coating and dip-coating are common methods for dispersing dyes into a polymer matrix due to their convenience and safety [12,13]. However, these solution processes cause dye aggregation or phase separation during or after drying that can lead to serious defects in the electro-optical properties [14]. The uniform dispersion of dyes and pigments into a polymer matrix is a key process for the fabrication of optoelectronic devices.

Dyestuffs are substances that have the dyeing capacity of fibers and films. When the dyestuffs and fibers or films have a chemical affinity for each other, the dyestuffs can easily penetrate the fibers or films. Dyeing is a useful tool to uniformly color fibers and films. The coloring process, in which the dyes are allowed to uniformly penetrate fibers and films, is performed by immersing the dyes in a liquid solution dispersed with dyestuffs. Since the fibers and films are colored by this technique to impregnate the dye itself with a dye, there is no interlayer peeling at the interface, as with a coating. Therefore, a stabilized functional fiber without dye aggregation or phase separation can possibly be obtained.

Highly transparent films can be prepared from unsaturated polyester (UP) resins. The application process of the UP resins before curing can be accomplished in a number of convenient processes such as spraying, deposition with an applicator, dipping, or melt-press.

In this study, we focused on the dyeing process using aqueous dispersion; then, we introduced a typical azo-carbazole dye, CACzE, to large size UP resin films, and, finally, evaluated the dyeing properties of the dye-doped UP resin films and their dynamic holographic properties.

## 2. Experimental Section

**Figure 1:**
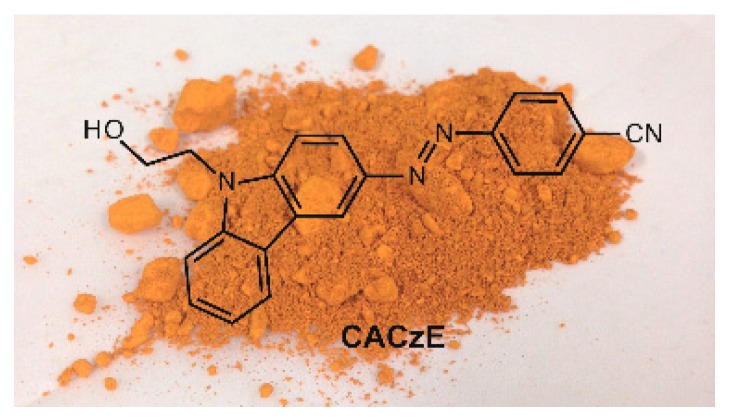
Structural formula of CACzE and background photograph of CACzE powder.

**Figure 2:**
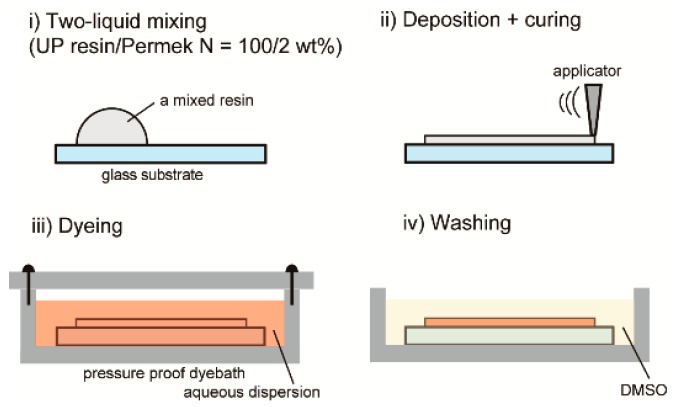
Schematic of the experimental procedure for the dispersion dyeing method. (**i**) A two-liquid mixing resin (UP resin/Permek N = 100/2 wt.%) deposited on a glass substrate; (**ii**) coating with an applicator with a 100 μm gap and curing; (**iii**) dyeing in the pressure proof dyebath; and (**iv**) washing with DMSO.

The 3-[(4-cyanophenyl)azo]-9*H*-carbazole-9-ethanol (CACzE) was synthesized according to a previously reported procedure [3]. Figure 1 shows the structural formula of CACzE and a photograph of the CACzE powder. The UP resin, as a host polymeric resin, and a curing agent, Permec N, which contains ethyl methyl ketone peroxides, dimethyl phthalate, and ethyl methyl ketone, were purchased from FRP-ZONE Co., Saitama, Japan. The surfactant polyoxyethylene (5) docosyl ether was purchased from Wako Co., Osaka, Japan. The mixtures containing 98% UP resin and 2% curing agent were vigorously stirred and deposited on a glass substrate by using a coating applicator with a 100 μm gap. The aqueous dispersion dyebaths were made via the dispersion of CACzE portions into 100 g of distilled water to provide 1.9 × 10^−4^ to 7.6 × 10^−3^ mol L^−1^ solutions (referred to herein as the aqueous dispersion). Additionally, specific amounts of the surfactant were added to the aqueous dispersion to adsorb the dispersed CACzE into the UP resin after curing. A summary of the aqueous dispersions is listed in Table 1. During the aqueous dispersion treatment process, the cured UP resins were immersed in the aqueous dispersion and shaken at 120 °C for various immersion cycles. The UP resins dyed with the aqueous dispersion were washed with dimethyl sulfoxide (DMSO) and air dried. Schematic diagrams of each process are shown in Figure 2.

**Figure 3:**
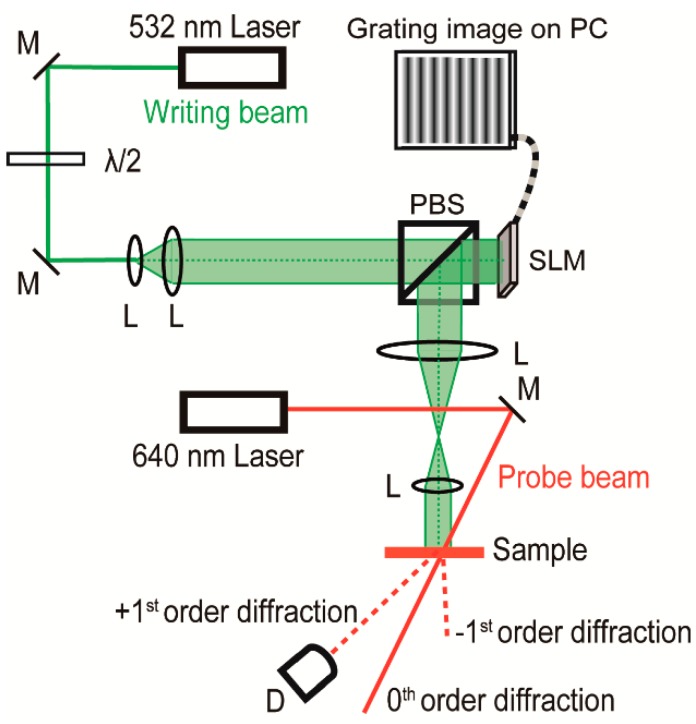
Schematic representation of the 4f reduction projection system: L—lens, M—mirror; PBS—polarizing beam splitter, PC—personal computer SLM—spatial light modulator, and D—photodiode. Laser sources are a green CW laser at 532 nm for recording and a red laser at 640 nm for reading.

The concentration of CACzE in the UP resin was recorded on a spectrophotometer (Lambda 1050 UV/Vis/NIR, Perkin Elmer, Waltham, MA, USA) and a laser Raman microscope (Raman-11, Nanophoton, Osaka, Japan) at room temperature. The Raman spectra were recorded under a 785 nm laser excitation. Transmittance spectra of the UP resin during the crosslinking reaction were measured by a Fourier Transform-Infrared Spectroscopy (FT-IR) spectrophotometer (FT/IR-4700 with ATR PRO ONE equipped with a diamond prism, Jasco, Tokyo, Japan). The ATR FT-IR spectrophotometer with a resolution of 1 cm^−1^ in the transmission mode was used for kinetic measurement of the crosslinking reaction. The glass-transition temperature (*T_g_*) was determined by a differential scanning calorimetry (DSC) (DSC2920, TA Instruments Co., New Castle, DE, USA) at a heating rate of 10 °C min^−1^. The haze value was measured to evaluate the transparency and scattering properties of the sample films using an integrating sphere. The haze value (%) was measured using an integral sphere and calculated as the total light intensity of the scattered light divided by the total light intensity of the sum of scattered and transmitted light. The geometry of the haze value measurement system is shown in Figure S1. The external diffraction efficiency was measured using a 4f reduction projection system, as shown in Figure 3. A vertical fringe pattern image with an *s*-polarized 532 nm writing beam reflected through a polarizing beam-splitter was projected directly onto the sample. A PC-controlled vertical fringe interference pattern with a grating number of 100 on a special light modulator (SLM) (1920 pixels wide × 1080 pixel high; 8.0 μm pixel size, HOLOEYE Photonics Co., Pittsfield, MA, Germany) provided a fringe pattern spacing of *Λ* = 25 μm on the sample surface. A weak *s*-polarized reading beam of 1 mW with a DPSS laser at 640 nm (Bolero^TM^, Cobolt Co., Solna, Sweden) was illuminated on the sample surface, and the first-order diffraction intensity from the resultant refractive index gratings was measured by a silicon photodiode.

We used the intensities of incident light (*I*_0_) and first-order diffracted light (*I_d_*_1_) to evaluate the first-order diffraction efficiency *η*_1_ with Equation (1):

(1) $$\eta_{1}=\frac{I_{d1}}{I_{0}}\times 100.$$

The response time *τ* of the first-order diffraction efficiency *η*_1_ as a function of time was fitted by the Kohlrausch-Williams-Watts stretched exponential function in Equation (2),

(2) $$\eta_{1}=\eta_{0}\left\{1-\exp\left[-{\left(\frac{t}{\tau }\right)}^{\beta }\right]\right\},$$

where *t* is the time, *η*_0_ is the steady-state external diffraction efficiency, and *β* (0 < *β* ≤ 1) is the parameter related to a dispersion.

## 3. Results and Discussion

**Figure 4:**
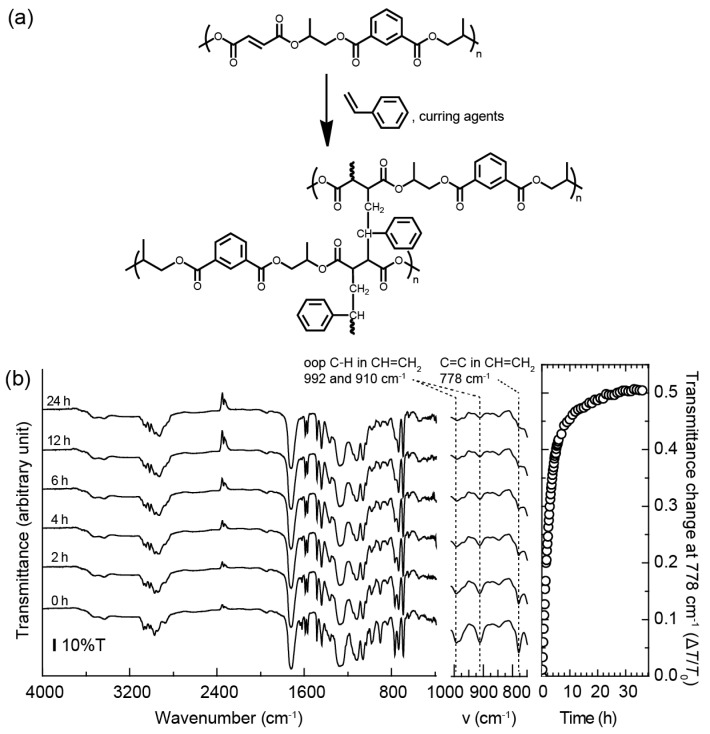
(**a**) Crosslinking reaction of the UP resin. (**b**) ATR-FT/IR spectra of a UP resin film and the transmittance change Δ*T*/*T*_0_ at 778 cm^−1^.

The UP resin was synthesized using the free radical chain-growth crosslinking reaction of an unsaturated polyester and styrene monomer with curing reagents, as shown in Figure 4a. During the crosslinking reaction at room temperature, the peroxides in the curing reagents acted as catalysts. The qualitative degree of the crosslinking in the UP resin containing 2 wt.% curing reagents was investigated using the ATR-FT/IR absorption measurements and analysis. Figure 4b shows the ATR-FT/IR absorption spectra for the UP resin after the addition of the curing reagents in the film. These spectra were measured in the dark, over time, at room temperature, and their spectra at each elapsed time were used for the kinetic evaluation of the crosslinking reaction. The ATR FT-IR spectrum of the as-prepared UP resin film shows absorptions at 3081, 3059, 3026, 2982, 2955, 1728, 1646, 1630, 1600, 1579, 1494, 1449, 1371, 1286, 1127, 1071,1042, 1021, 992, 910, 846, 778, 743, and 701 cm^−1^. These bands are ascribed to the terminal methylene C–H stretching (3081 cm^−1^), aromatic C–H stretching (3059 and 3026 cm^−1^), symmetric C–H stretching 2982 cm^−1^, aliphatic C–H stretching (2955 cm^−1^), C=O stretching (1728 cm^−1^), C=C stretching (1646 and 1630 cm^−1^), aromatic C=C stretching (1600 and 1579 cm^−1^), C–H stretching (1494 cm^−1^), C–H bending (1449 cm^−1^), aliphatic C–H stretching in methyl (1371 cm^−1^), Ph–C=O stretching (1286 cm^−1^), Ph–C–O stretching (1127 cm^−1^), C–H in-plane deformation (1071 cm^−1^), out-of-plane C–H bending in CH=CH_2_ (992 and 910 cm^−1^), C–O–C stretching (846 cm^−1^), C=C stretching in CH=CH_2_ (778 cm^−1^), and aromatic out-of-plane C–H bending (743 and 701 cm^−1^) of the UP resin and styrene. The transmittances based on the absorption of C=C stretching at 1646 and 910 cm^−1^, out-of-plane C–H bending in CH=CH_2_ at 992 and 910 cm^−1^, and C=C stretching in CH=CH_2_ at 778 cm^−1^ are significantly increased upon increase of the curing time, implying the reduction of the C=C bonds is due to the progress of the crosslinking reaction at room temperature. The reduction of the C=C in styrene during the crosslinking reaction was determined by the transmittance change, Δ*T*/*T*_0_ (Δ*T* is an absolute change in the transmittance given by *T*−*T*_0_, where *T* is the transmittance at a time after curing and *T*_0_ is the transmittance before curing), at 778 cm^−1^. The plots of the transmittance change Δ*T*/*T*_0_ leveled out at approximately 24 h curing, which indicated that the crosslinking reaction was almost complete at 24 h. It should be noted here that most of the styrene monomer does not remain in the UP resin film after curing for 24 h. Because the reaction between the styrene monomer and fumaric acid ester is faster than the reaction between styrene monomers, it is assumed that styrene polymer does not remain in the UP resin film. As shown in Figure S2, the glass-transition temperature (~100 °C.) of the styrene polymer was not detected. The glass-transition temperature, *T*_g_, of the UP resin film after curing for 24 h was 33.2 °C. This glass-transition temperature of the UP resin film indicated that the amount of styrene contained in the UP would be 6% or less [15]; it has been reported that the UP resin is a stable film without weight loss up to about 300 °C. In addition, swelling and shrinkage before and after curing of the UP resin were not confirmed. Descriptions of the complicated curing mechanisms of the UP resin have been extensively reported in the literature [16]. Optical transparency is one of the most important characteristics of the holographic display. The haze value (%), defined by the ratio of diffuse transmittance (%)/total light transmittance (%) × 100, was measured for the UP resin film after curing and before dyeing, and was found to be 5.0%. Although a haze value of 4% or below is prefereable for holographic display applications, the haze values are acceptable for reflection and transmission holography. Furthermore, an UP resin film with a haze value of 5% is a smooth surface and shows high transparency with no scattering; this value is similar to the polymethyl methacrylate (PMMA) haze value of 2.6% [7]. The evaluation of the haze value for the obtained UP resin film is useful for the performance of holograms and other research fields, such as organic light emitting devices.

**Figure 5:**
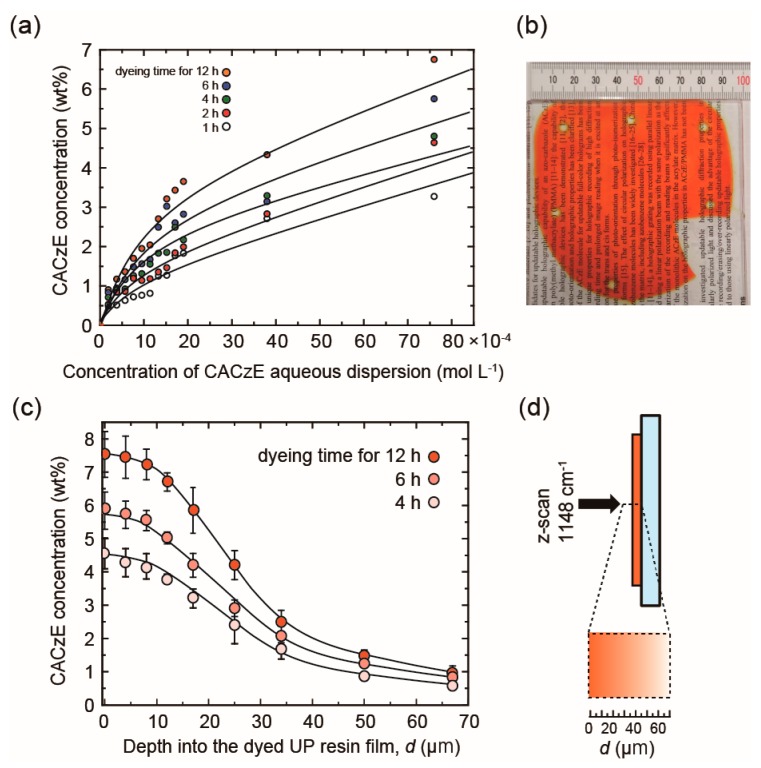
(**a**) Dye uptake isotherms of the CACzE dye on the UP resin film (dyebath temp. 120 °C); (**b**) a photograph of an UP resin film with 100 mm square after dyeing; (**c**) CACzE concentration vs. depth profiles for the dye-doped UP resin films (Entry 10); and (**d**) a schematic illustration of dye-doped UP resin film.

The UP resin films are immersed in the aqueous dispersion dyebath and heated at a temperature above the *T*_g_ of the UP resin before the CACzE dyes are absorbed into the UP resin film. The CACzE dyes that remain at the surface of the UP resin films are removed with DMSO. The concentration of the CACzE dye uptake in the UP resin films was estimated based on the absorbance at 561 nm, as shown in Figure S3. The concentration of the CACzE dye uptake increased from 0.49 to 6.75 wt.% by increasing the concentration of the aqueous dispersion and the immersion time (all results are summarized in Table S1). The relationship between the equilibrium of CACzE dye uptake and the concentration of CACzE aqueous solution is shown in Figure 5a, and the Freundlich isotherm model was applied to the equilibrium data. For ordinary adsorption, the Langmuir adsorption isotherm model, showing a saturation point with increasing concentrations, should be adopted. However, it is suggested that the adsorption curves of the UP resin films appear not to be saturated; that is, the Freundlich isotherm model, which fits well with the adsorption behavior in the low concentration region, would be appropriate [17]. As a result, the maximum concentration of the CACzE dye uptake in the UP resin film was 6.75 wt.% at 7.60 × 10^−3^ mol L^−1^ CACzE aqueous dispersion (Entry 12), with a dyebath temperature at 120 °C, and an immersion time of 12 h. When the concentration, immersion time or dyebath temperature were higher than these conditions, which demonstrated the maximum dye uptake, the UP resin film resulted in more light scattering and brittle texture. Figure 5b shows a photograph of a 100 mm square size UP resin film with a dye uptake of 3.65 wt.% after dyeing (Entry 10, immersion time 12 h). The large size UP resin film was uniformly dyed, and its transparency was remained relatively high, showing a smooth surface; however, the haze value was 11.6%. Figure 5c shows the depth profiles of the dye-doped UP resin film (Entry 10), where the Raman peak of Ph–N= [18] unit in CACzE dye at 1148 cm^−1^ was measured using laser Raman spectroscopy, and are plotted as a function of the measurement depth. Figure 5d shows a schematic illustration for the laser Raman measurement and the schematic profile of dye concentration distribution in the direction of film depth. The CACzE dye concentration vs. the depth profile shows a diffusion-controlled distribution of the CACzE dye concentration, which indicates that the CACzE concentration declines as the depth into the film bulk increases. The average concentration of the CACzE dye uptake in the UP resin film dyed at 1.90 × 10^−3^ mol L^−1^ CACzE aqueous dispersion (Entry 10), a dyebath temperature at 120 °C, and the immersion time for 12 h was 3.65 wt.% based on the absorbance at 561 nm. On the other hand, as shown in Figure 5c, the concentration of the CACzE dye in the range of 10 μm from the surface was 7.53 wt.%; however, the CACzE dye concentration significantly decreased at the deeper position in the film. This dyeing process is a noteworthy technique for fabricating a ~10 μm thick film with a dye concentration of 7 wt.% or higher, which can be much more powerful and effective for large size film than the spin-coating technique. Furthermore, the advantage of the dyeing process is that it is not a batch process and therefore has a relatively high throughput compared to the spin-coating process. Finally, the actual amount of dye used in the dyeing process was very low, and it was possible to incorporate all of dye into the substrate. The dyeing process will certainly be effective for holographic application requiring large area transparency. The depth profiles are noted; the CACzE concentration is near zero at a depth of 70 μm, and a film thickness of 70 μm or less is preferable in this dyeing process.

The holographic gratings for the films containing azobenzene moieties were induced by two kinds of processes; a modulation of the polarization grating due to the nanoscopic angular reorientation of azobenzene moieties and a modulation of the surface relief grating induced by a macroscopic molecular migration of azobenzene molecules. The latter is well-known to produce thermally stable gratings [19]. In the present case, no surface relief gratings were observed on the surface of the dye-doped UP resin films.

**Figure 6:**
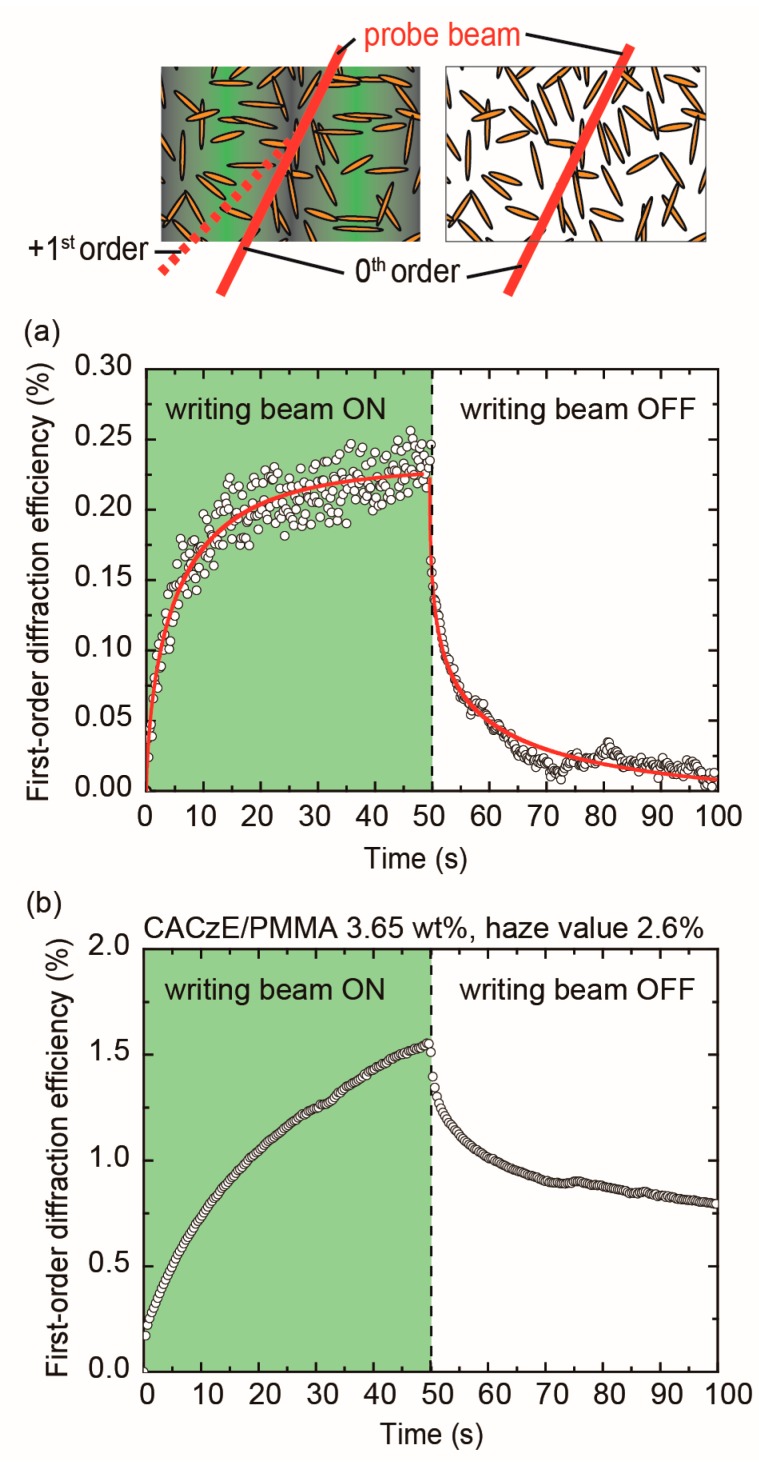
Top-view schematic representation of diffraction responses of probe beam for perpendicular orientation (left) and random orientation (right) of the CACzE dyes. (**a**) Time evolution of the first-order diffraction efficiency for the dye-doped UP resin film with 3.65 wt.% (Entry 10, immersion time 12 h). (**b**) Time evolution of the first-order diffraction efficiency for the melt-press CACzE/PMMA with 3.65 wt.%.

The first order diffraction efficiency, *η*_1_, was plotted as a function of the recording time, followed by the elapsed time at room temperature for the dye-doped UP resin film (Figure 6). The interference fringe pattern of the writing beam was turned on at time zero. As the time elapsed, the increase in the first-order diffraction efficiency *η*_1_ was measured. The writing beam of the interference fringe pattern was turned off at 50 s. As a result, a steady-state *η*_1_ of 0.23%, a response (rising) time *τ* of 5.9 s, and a decay time of 3.6 s were measured. In comparison, a melt-pressed PMMA film containing CACzE of 3.65 wt.% (CACzE/PMMA), and a first-order diffraction efficiency *η*_1_ at 5.9 s was almost the same value (*η*_1_ = 0.55%); however, the haze value of the dye-doped UP resin film was slightly higher than the value of the CACzE/PMMA of 3.65 wt.%. Therefore, the optical loss seemed to be lowering the first-order diffraction efficiency of the dye-doped UP resin film. The diffraction grating can be classified as the Raman-Nath regime because the product of the grating thickness *d* and the writing beam wavelength *λ* in the film was smaller than the square of the fringe pattern spacing *Λ*, *Λ*^2^ > *dλ*. The equation for the theoretical grating thickness *d*_t_ is *I* = *I*_0_ exp(−*αd*_t_), where an absorption coefficient *α* = 126 cm^−1^ at 532 nm, and it is estimated to be 365 μm. The absorption coefficient *α* of the dyed-doped UP resin film was given by absorption spectral feature (Figure S3, Supplementary Materials). Accordingly, *Λ*^2^ > *dλ* held, and the diffraction grating could be determined as the Raman-Nath regime. The result indicated that it was clearly different from the azo-mesogenic polymers or azo-elastomers showing Bragg diffraction derived from the surface relief grating which has been reported so far [20,21,22,23] The refractive index modulation Δ*n* in a transmitted Raman-Nath grating is proportional to the diffraction efficiency for sinusoidal phase grating expressed by first-order Bessel functions [24,25]:

(3) $$\eta_{1}=J_{1}^{2}(\delta )=J_{1}^{2}\left(\frac{2\pi d\Delta n}{\lambda \cos\theta }\right)$$

where *η*_1_ is the first-order diffraction efficiency, *J*_1_ is the first-order Bessel function, *d* is the grating thickness, Δ*n* is the refractive index modulation, *θ* is the incidence angle of the reading beam within the film, and *δ* is the Raman-Nath parameter. Thus, a refractive index modulation Δ*n* was estimated to be 1.3 × 10^−4^ with first-order diffraction efficiency of 0.23% and the other parameters were: *θ* = 21.5°, *λ* = 640 nm, *δ* = 0.096, and *d* = 70 μm. The grating thickness *d* considered here corresponds to the measured depth profile of the CACzE concentration. However, the refractive index modulation Δ*n* of 1.3 × 10^−4^, corresponding to the steady-state *η*_1_ of 0.23%, showed a relatively low value compared to the values previously reported [7]. The cause of this low diffraction efficiency in the film is established in Figure 5c. The concentration of dyes had a significant gradient along the depth into the UP resin film. This concentration gradient of the CACzE dyes may be responsible for the low optical diffraction. Another possible reason is the difference of the matrix. The former matrix was poly(methyl methacrylate) (PMMA) and the present matrix is epoxy resin. The environment around the CACzE dyes in the matrix significantly affect the photo-isomerization process. For example, the interaction between the CACzE dyes and matrix and/or the free volume in the matrix allowing the rotation of the trans-cis photo-isomerization would affect the optical diffraction. These points should be clarified in future studies. However, the present results of the diffraction properties in the dye-doped UP resin film provide the success of the holographic film using the dyeing process.

The response times of the dye-doped UP resin film show a faster response than those of the melt-pressed CACzE/PMMA film (the response time is not saturated within 50 s as shown in Figure 6b), which may be due to the higher glass-transition temperature *T*_g_ of the CACzE/PMMA film than the dye-doped UP resin film. The thermal decay of *η*_1_ for the dye-doped UP resin film after being turned off is shown in Figure 6a. The decay time corresponded to the single exponential decay function, and the decay time constant *τ*_d_ was estimated to be 3.6 s, faster than that of the melt-pressed CACzE/PMMA film. The holographic properties of the dye-doped UP resin film obtained in the dyeing process were relatively low in comparison to a standard melt-pressed PMMA film containing 30 wt.% CACzE dye; however, the film size was 16 times larger than the standard film. In other words, if a large size, as well as high concentration, can be achieved by improving the dyeing process, a large size film with high holographic properties will be obtained.

In conclusion, the dyeing process successfully introduced the CACzE dyes into the UP resin film in an aqueous solution. The gradient concentration ranging from 6.75 to 0.49 wt.% was measured along the depth from the sample surface, and the concentration in the range of 10 μm from the surface was approximately twice as high as the average concentration of 3.65%. The typical holographic characteristics, including a steady-state *η*_1_ of 0.23%, response time *τ* of 5.9 s, and decay time of 3.6 s, are given.

## 4. Conclusions

The holographic properties of a CACzE azo-carbazole dye in a UP resin were investigated. The crosslinking of the UP resin containing 2 wt.% curing agents was evaluated using an ATR-FT/IR analysis. The CACzE dyes were successfully dispersed into the UP resin film in the aqueous dye solution. The total amount of dye uptake during the dyeing process ranged from 0.49 to 6.75 wt.%. The dye-doped UP resin films using the dyeing process showed that the resulting concentrations of the CACzE dye uptake increased in proportion to the concentration of the aqueous solution and the immersion time, ranging from 0.49 to 6.75 wt.%. The dye-doped UP resin film with 3.65 wt.% exhibited a steady-state holographic diffraction efficiency of *η*_1_ of 0.23%, response time of *τ* of 5.9 s, and decay time of 3.6 s. The present dyeing process using aqueous solutions is a contribution to, and an advantage for, fabricating large-sized holographic devices as well as fabricating the photonic devices based on any polymer film containing organic dye.

## Figures and Tables

**Table 1:** Experimental conditions for the aqueous dispersions.

Entry	CACzE Aqueous Dispersion (10^−4^ mol L^−1^)	Surfactant (g)
1	1.9	0.1
2	3.8	0.2
3	5.7	0.3
4	7.6	0.4
5	9.5	0.5
6	11.4	0.6
7	13.3	0.7
8	15.2	0.8
9	17.1	0.9
10	19.0	1.0
11	38.0	2.0
12	76.0	4.0

## Supplementary Materials

The following are available online at https://www.mdpi.com/1996-1944/12/6/945/s1, Figure S1: Haze value measurement system. (a) Measuring total transmitted light intensity. (b) Measuring scattered transmitted light intensity. A collimated light of 636 nm was used as the probe beam. Figure S2: DSC thermogram with heat flow signal vs. temperature for the UP resin film after curing for 24 h. Figure S3: UV–visible absorption spectra of the dyed UP resin films at each immersion time. Table S1: Film thickness, absorbance, dye uptake for the UP resin films after dyeing processes.
