# Supplementary material for: Holographic Performance of Azo-Carbazole Dye-Doped UP Resin Films Using a Dyeing Process
Source: Materials (Basel). 2019 Mar 21;12(6):945. doi: 10.3390/ma12060945 (PMC6470482; doi:10.3390/ma12060945)
Supplement: Supplementary file 1 [file materials-12-00945-s001.pdf]

materials

Supplementary Materials

Holographic Performance of Azo-Carbazole Dye-Doped UP Resin Films Using a Dyeing Process

Kenji Kinashi [1,*], Isana Ozeki [2], Ikumi Nakanishi [2], Wataru Sakai [1] and Naoto Tsutsumi [1,*]

1 Faculty of Materials Science and Engineering, Kyoto Institute of Technology, 606-8585 Kyoto, Japan; wsakai@kit.ac.jp
2 Master's Program of Innovative Materials, Graduate School of Science and Technology, Kyoto Institute of Technology, Matsugasaki, Sakyo, 606-8585 Kyoto, Japan; isana.oz.0611@gmail.com (I.O.); m7616023@edu.kit.ac.jp (I.N.)
* Correspondence: kinashi@kit.ac.jp (K.K.); tsutsumi@kit.ac.jp (N.T.)

Haze value measurement system

The haze value was measured to evaluate the transparency and scattering properties of the samples using an integrating sphere (Figure S1). The haze value (%) was calculated by

$$\text{Haze value } (\%) = \frac{T_d}{T_t} \times 100,$$

where T_t is the total transmitted light intensity, and T_d is the scattered transmitted light.

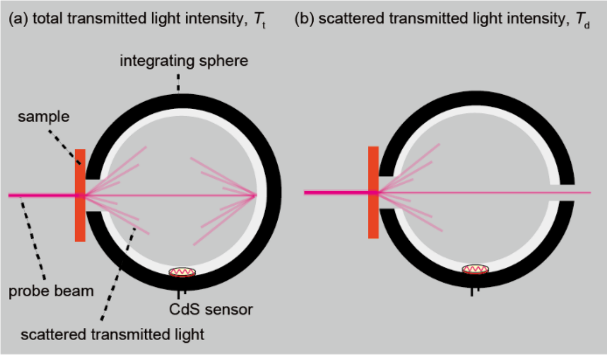

Figure S1. Haze value measurement system. (a) Measuring total transmitted light intensity. (b) Measuring scattered transmitted light intensity. A collimated light of 636 nm was used as the probe beam.

Differential Scanning Calorimetry (DSC)

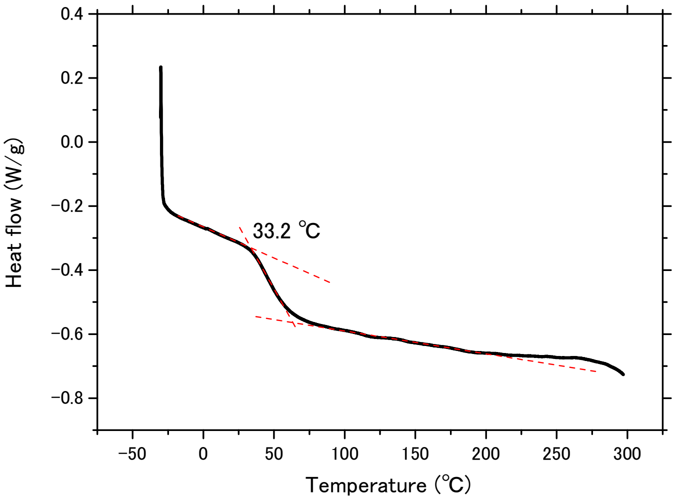

Figure S2. DSC thermogram with heat flow signal vs. temperature for the UP resin film after curing for 24 h.

Absorption Spectra

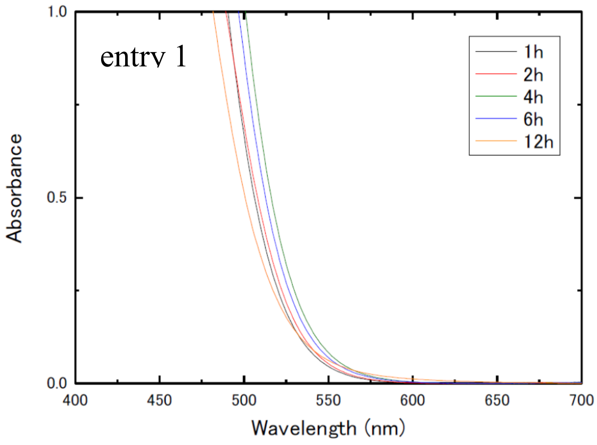

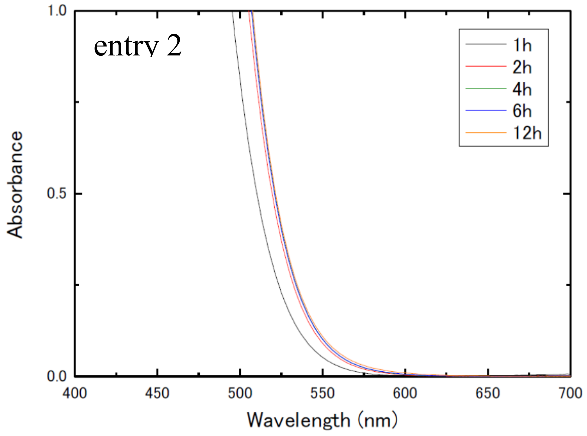

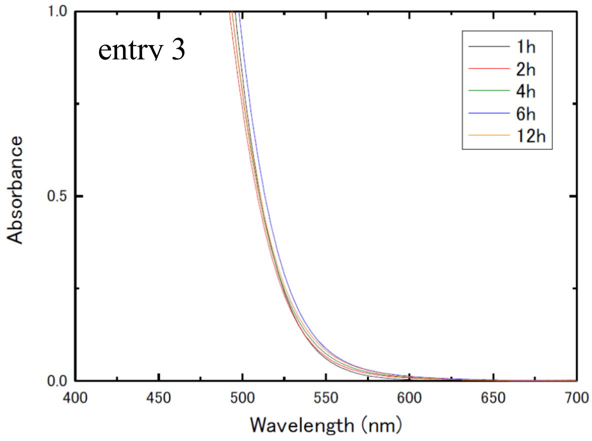

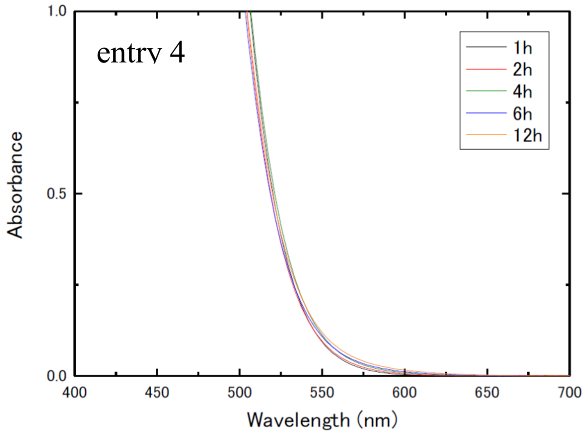

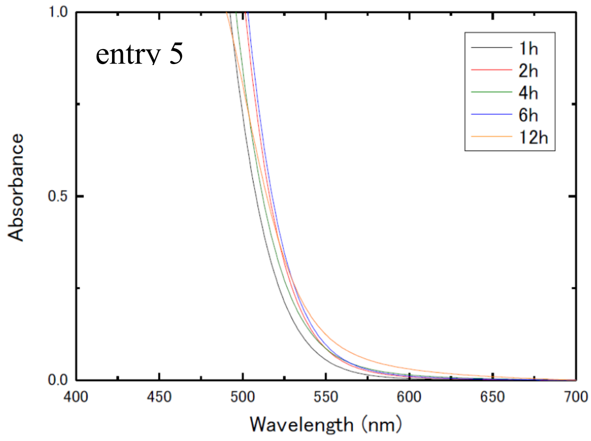

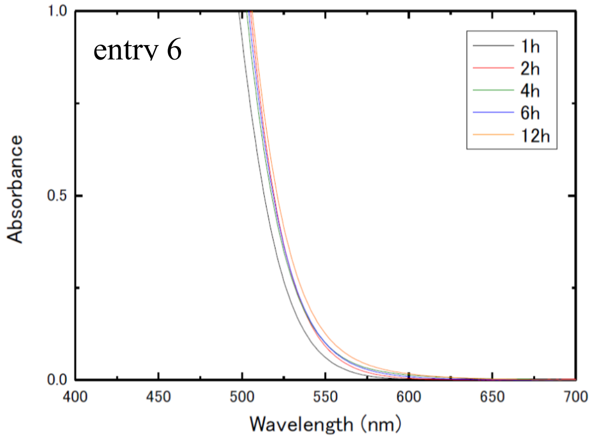

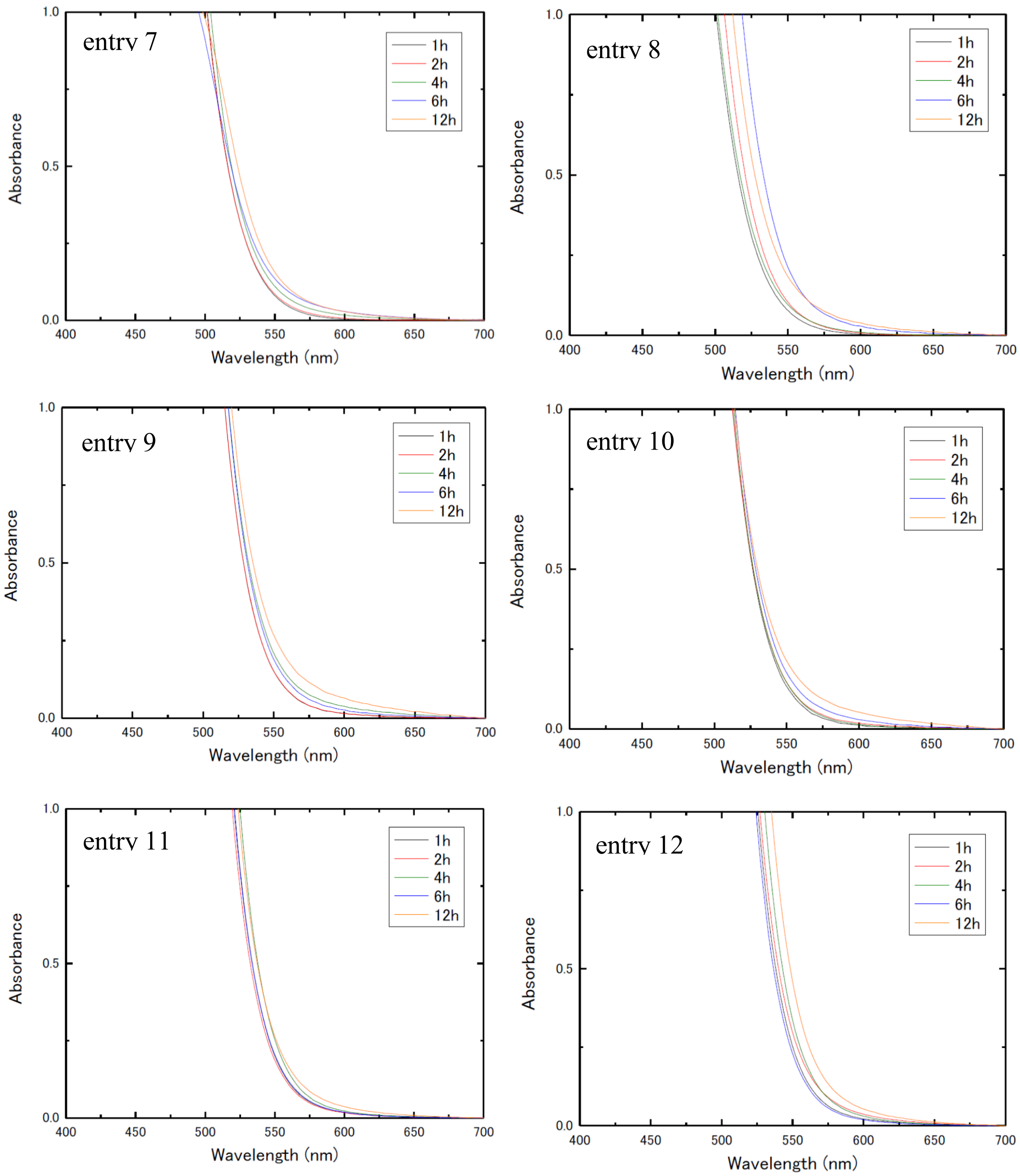

Figure S3. UV–visible absorption spectra of the dyed UP resin films at each immersion time.

Table S1. Film thickness, absorbance, dye uptake for the UP resin films after dyeing processes.

	immersion time (h)	1	2	4	6	12	24
entry 1	thickness (µm)	97	107	131	94	94	—[1)]
	absorbance at 561 nm	0.023	0.027	0.044	0.037	0.041	
	dye uptake (wt%)	0.49	0.53	0.71	0.83	0.90	
entry 2	thickness (µm)	103	135	126	121	109	—[1)]
	absorbance at 561 nm	0.025	0.047	0.053	0.054	0.061	
	dye uptake (wt%)	0.51	0.84	0.90	0.94	1.17	
entry 3	thickness (µm)	107	93	100	96	78	—[1)]
	absorbance at 561 nm	0.032	0.038	0.044	0.053	0.051	
	dye uptake (wt%)	0.63	0.86	0.92	1.16	1.36	
entry 4	thickness (µm)	139	78	104	90	90	—[1)]
	absorbance at 561 nm	0.048	0.044	0.063	0.064	0.073	
	dye uptake (wt%)	0.72	1.19	1.26	1.48	1.70	
entry 5	thickness (µm)	83	89	85	74	91	—[1)]
	absorbance at 561 nm	0.030	0.049	0.053	0.055	0.085	
	dye uptake (wt%)	0.76	1.14	1.31	1.56	1.95	
entry 6	thickness (µm)	78	85	80	71	78	—[1)]
	absorbance at 561 nm	0.030	0.048	0.059	0.057	0.076	
	dye uptake (wt%)	0.81	1.18	1.55	1.67	2.04	
entry 7	thickness (µm)	70	73	77	75	77	—[1)]
	absorbance at 561 nm	0.042	0.047	0.067	0.089	0.099	
	dye uptake (wt%)	1.25	1.35	1.83	2.49	2.70	
entry 8	thickness (µm)	68	82	67	86	79	—[1)]
	absorbance at 561 nm	0.041	0.057	0.059	0.124	0.121	
	dye uptake (wt%)	1.27	1.46	1.85	4.02	3.21	
entry 9	thickness (µm)	99	96	110	91	109	—[1)]
	absorbance at 561 nm	0.084	0.085	0.129	0.113	0.179	
	dye uptake (wt%)	1.77	1.84	2.46	2.60	3.43	
entry 10	thickness (µm)	82	87	78	80	82	—[1)]
	absorbance at 561 nm	0.072	0.083	0.081	0.108	0.143	
	dye uptake (wt%)	1.83	1.99	2.17	2.82	3.65	
entry 11	thickness (µm)	84	75	88	75	76	—[1)]
	absorbance at 561 nm	0.109	0.101	0.139	0.113	0.157	
	dye uptake (wt%)	2.71	2.83	3.29	3.14	4.33	
entry 12	thickness (µm)	86	77	80	67	80	—[1)]
	absorbance at 561 nm	0.134	0.171	0.184	0.184	0.258	
	dye uptake (wt%)	3.27	4.64	4.80	5.75	6.75	

[1)] Not measured

© 2019 by the authors. Licensee MDPI, Basel, Switzerland. This article is an open access article distributed under the terms and conditions of the Creative Commons Attribution (CC BY) license (http://creativecommons.org/licenses/by/4.0/).
